# 3T sodium-MRI as predictor of neurocognition in nondemented older adults: a cross sectional study

**DOI:** 10.1093/braincomms/fcae307

**Published:** 2024-09-11

**Authors:** Elaine Lui, Vijay K Venkatraman, Sue Finch, Michelle Chua, Tie-Qiang Li, Bradley P Sutton, Christopher E Steward, Bradford Moffat, Elizabeth V Cyarto, Kathryn A Ellis, Christopher C Rowe, Colin L Masters, Nicola T Lautenschlager, Patricia M Desmond

**Affiliations:** Department of Radiology, The University of Melbourne, Parkville, 3050 Victoria, Australia; Department of Medical Imaging, The Royal Melbourne Hospital, Parkville, 3050 Victoria, Australia; Department of Radiology, The University of Melbourne, Parkville, 3050 Victoria, Australia; Department of Medical Imaging, The Royal Melbourne Hospital, Parkville, 3050 Victoria, Australia; Statistical Consulting Centre, University of Melbourne, Parkville, 3010 Victoria, Australia; Department of Medical Imaging, The Royal Melbourne Hospital, Parkville, 3050 Victoria, Australia; Department of Clinical Science, Intervention and Technology, Karolinska Institute, 171 77 Stockholm, Sweden; Beckman Institute for Advance Science and Technology, University of Illinois at Urbana Champaign, Champaign, IL 61801, USA; Department of Bioengineering, University of Illinois at Urbana-Champaign, Champaign, IL 61820, USA; Department of Radiology, The University of Melbourne, Parkville, 3050 Victoria, Australia; Department of Medical Imaging, The Royal Melbourne Hospital, Parkville, 3050 Victoria, Australia; Department of Radiology, The University of Melbourne, Parkville, 3050 Victoria, Australia; Australian Centre for Health Services Innovation and Centre for Healthcare Transformation, School of Public Health and Social Work, Faculty of Health, Queensland University of Technology, Brisbane, Queensland 4059, Australia; Academic Unit for Psychiatry of Old Age, Department of Psychiatry, The University of Melbourne, Melbourne, 3010 Victoria, Australia; Melbourne School of Psychological Sciences, University of Melbourne, Melbourne, 3010 Victoria, Australia; Department of Molecular Imaging and Therapy, Austin Health, Melbourne, 3084 Victoria, Australia; The Florey Institute of Neuroscience and Mental Health, The University of Melbourne, Melbourne, 3052 Victoria, Australia; The Florey Institute of Neuroscience and Mental Health, The University of Melbourne, Melbourne, 3052 Victoria, Australia; Academic Unit for Psychiatry of Old Age, Department of Psychiatry, The University of Melbourne, Melbourne, 3010 Victoria, Australia; Royal Melbourne Hospital Mental Health Service, Royal Melbourne Hospital, Parkville, Melbourne, 3052 Victoria, Australia; Department of Radiology, The University of Melbourne, Parkville, 3050 Victoria, Australia; Department of Medical Imaging, The Royal Melbourne Hospital, Parkville, 3050 Victoria, Australia

**Keywords:** neurocognition, sodium-MRI, 3-Tesla, nondemented

## Abstract

Dementia is a burgeoning global problem. Novel magnetic resonance imaging (MRI) metrics beyond volumetry may bring new insight and aid clinical trial evaluation of interventions early in the Alzheimer’s disease course to complement existing imaging and clinical metrics. To determine whether: (i) normalized regional sodium-MRI values (Na-SI) are better predictors of neurocognitive status than volumetry (ii) cerebral amyloid PET status improves modelling. Nondemented older adult (>60 years) volunteers of known Alzheimer's Disease Assessment Scale (ADAS-Cog11), Mini-Mental State Examination (MMSE) and Consortium to Establish a Registry for Alzheimer's Disease (CERAD) neurocognitive test scores, ApolipoproteinE (APOE) e4 +/− cerebral amyloid PET status were prospectively recruited for 3T sodium-MRI brain scans. Left and right hippocampal, entorhinal and precuneus volumes and Na-SI (using the proportional intensity scaling normalization method with field inhomogeneity and partial volume corrections) were obtained after segmentation and co-registration of 3D-T1-weighted proton images. Descriptive statistics, correlation and best-subset regression analyses were performed. In our 76 nondemented participants (mean(standard deviation) age 75(5) years; woman 47(62%); cognitively unimpaired 54/76(71%), mildly cognitively impaired 22/76(29%)), left hippocampal Na-SI, not volume, was preferentially in the best models for predicting MMSE (Odds Ratio (OR) = 0.19(Confidence Interval (CI) = 0.07,0.53), *P*-value = 0.001) and ADAS-Cog11 (Beta(B) = 1.2(CI = 0.28,2.1), *P*-value = 0.01) scores. In the entorhinal analysis, right entorhinal Na-SI, not volume, was preferentially selected in the best model for predicting ADAS-Cog11 (B = 0.94(CI = 0.11,1.8), *P*-value = 0.03). While right entorhinal Na-SI and volume were both selected for MMSE modelling (Na-SI OR = 0.23(CI = 0.09,0.6), *P*-value = 0.003; volume OR = 2.6(CI = 1.0,6.6), *P*-value = 0.04), independently, Na-SI explained more of the variance (Na-SI *R*^2^ = 10.3; volume *R*^2^ = 7.5). No imaging variable was selected in the best CERAD models. Adding cerebral amyloid status improved model fit (Akaike Information Criterion increased 2.0 for all models, *P*-value < 0.001–0.045). Regional Na-SI were more predictive of MMSE and ADAS-Cog11 scores in our nondemented older adult cohort than volume, hippocampal more robust than entorhinal region of interest. Positive amyloid status slightly further improved model fit.

## Teaser

Lui & Venkatraman *et al.* report that normalized hippocampal sodium MRI signal were more predictive of Alzheimer's Disease Assessment Scale Cognitive subscale and Mini-Mental State Examination scores in a nondemented older adult cohort than hippocampal cortical volumes and conclude that sodium MRI may have applications as an imaging neurocognitive marker.

## Graphical Abstract

**Graphical Abstract:**
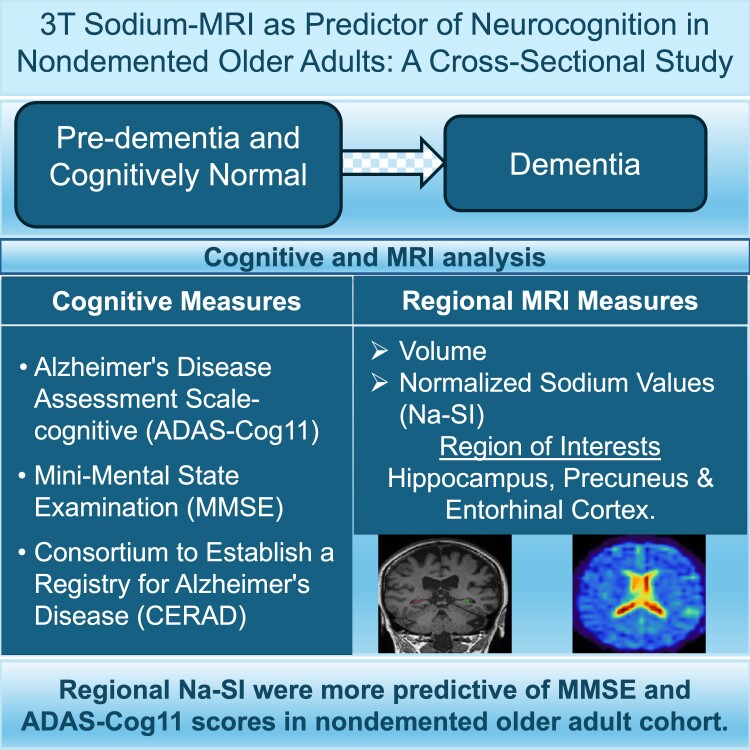

## Introduction

Dementia is a burgeoning global problem. Alzheimer’s disease (Ad) is predicted to increase to 75 million globally and associated healthcare cost to rise to US$2 trillion by 2030.^1^ Therapeutic options remain limited, with a historically dismal drug development failure rate of over 99%.^2^ Aducanumab and Lecanemab have received accelerated FDA approval for Ad as anti-amyloid disease-modifying therapy (DMT) agents. However, more data on clinical efficacy through a demonstratable slowing of cognitive decline (beyond surrogate neuropathologic endpoints such as amyloid clearance) is needed to non-controversially justify the widespread use of these DMT.^3,4^

MRI is used to monitor side effects in DMT clinical trials, such as amyloid-related imaging abnormalities, while MR hippocampal volumetry is usually included as a secondary outcome.^5,6^ However, atrophy is relatively late along the Ad spectrum.^7^ With DMT targeting the pre-clinical to early phases of the Ad spectrum,^3,5,6^ a sufficiently sensitive MRI metric predictive of neurocognitive status in this target group would be invaluable, with potential to reduce duration, size and cost of trials and increase evidence of efficacy to accelerate and better inform trial and regulatory decisions.

Sodium-MRI is a candidate with potential to fulfill this need. Sodium ion concentrations are compartmentalized and tightly regulated in normal cell homeostasis; the transmembrane gradient is actively maintained by the highly energy-consuming sodium-potassium ATPase. Cell death and resulting loss of cell membrane integrity, sodium-potassium pump dysfunction, sodium channel up/downregulation, other interlinked membrane or ionic/metabolic alterations (such as calcium^8^), and extracellular changes all affect brain tissue sodium values. Preclinical studies also support a role of these metabolic perturbations in the pathophysiology of AD.^9-12^ In-vivo human sodium-MRI brain studies have furthermore shown differences between control and Ad groups,^13-16^ stable sodium values across normal ageing,^17^ and differences in functional disability metrics for Alzheimer’s disease and other neurodegenerative conditions compared to controls.^15,16,18,19^ Sodium-MRI has also been shown to be predictive of Montreal Cognitive Assessment scores in AD.^15,16^

Would sodium-MRI likewise be predictive of neurocognitive test scores in a spectrum of nondemented older adults, the target group for dementia DMT trials? We hypothesized, if tissue sodium is a more sensitive metric of early neuronal dysfunction and neurodegeneration than atrophy, that normalized sodium-MRI values (Na-SI)—normalization is a procedure that uses the SI of a reference region as denominator to allow for interscan SI comparisons^13,20^—would also be a better predictor of neurocognition than volume across the spectrum of nondemented older adults.

Therefore, in this study, we aimed to evaluate whether regional Na-SI values are better predictors of neurocognitive test scores than their respective volumes. As we are exploring our hypothesis in the context of potential application in Ad trials, we assessed three regions and three tests relevant to Ad and we also secondarily aimed to evaluate whether including cerebral amyloid PET status would improve score prediction.

## Materials and methods

### Participants

English-speaking adults older than 60 years from the community were prospectively recruited after informed consent and institutional ethics approval. Nondemented individuals from the Melbourne arm of the Australian Imaging, Biomarkers and Lifestyle (AIBL) study^21^ who responded to our invitation to participate in this sodium MRI sub-study were enrolled. Their nondemented status was assigned by consensus from a panel of geriatric psychiatrists, neurologists, geriatricians and neuropsychologists according to internationally agreed criteria^22,23^ after clinical review and neuropsychological testing, which included ADAS-Cog11, MMSE and CERAD-Praxis (higher score = worse cognition for ADAS-Cog11, lower score = worse cognition for MMSE and CERAD). Age, sex, education level, APOE e4 carrier status, ADAS-Cog11, MMSE and CERAD scores, and cerebral amyloid PET status at the sodium-MRI scan timepoint were collected as part of the AIBL and AIBL-Active projects.^21,24^

Exclusion criteria included contraindication to MRI, dementia, schizophrenia, bipolar disorder, depression, Parkinson’s disease, cancer, symptomatic stroke, uncontrolled diabetes, and excessive alcohol use (>2 and 4 standard drinks/day for women and men respectively).

### MRI acquisition

MRI was performed on a 3T scanner (Trio, Siemens, Erlangen, Germany). Sodium images were acquired using the flexible twisted projection imaging sequence^25^ (TR = 160 ms, TE = 0.3 ms, flip angle = 90 degrees, averages = 2 at 5 mm isotropic resolution) using a 123.2/32.6 MHz sodium-proton dual-tuned quadrature transmit/receive head coil (Rapid Biomedical, Rimpar, Germany). 3D-T1 images were obtained using a 12-channel proton head coil in the same imaging session (TR = 1900ms, TE = 2.13 ms, FA = 9 degrees at 1 mm isotropic resolution). Additional T2 images (TR = 3000 ms, TE = 98 ms, FA = 120 degrees at 0.43mm*0.43mm*3 mm resolution) were obtained to improve proton-sodium image co-registration, and two other flexible twisted projection imaging sodium acquisitions were made, one with TE = 2.6 ms for B0 inhomogeneity correction, the other at flip angle = 45 degrees for B1 inhomogeneity correction.^25^

### Imaging data processing and analysis

**Figure 1:**
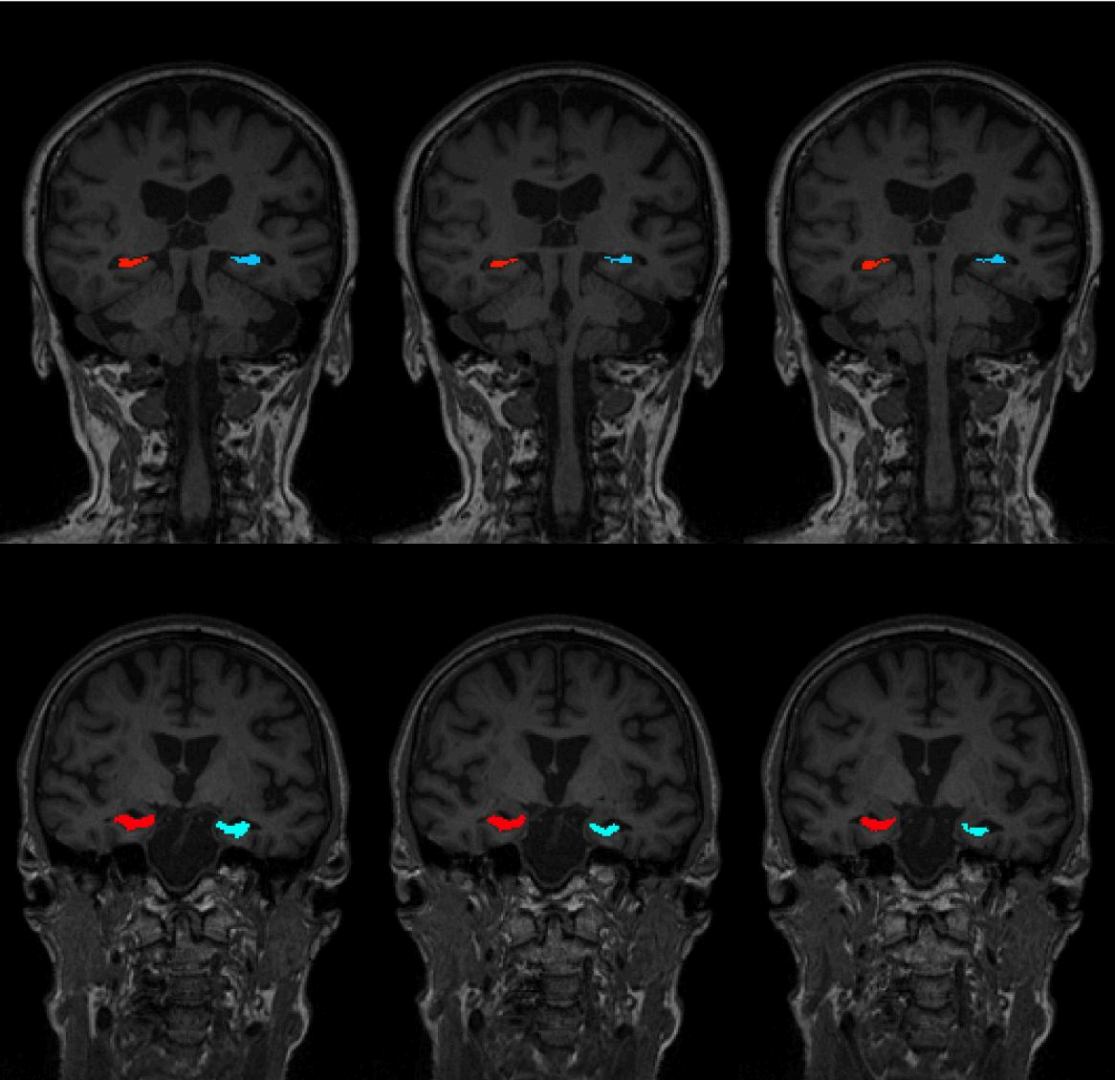
**Example of hippocampal segmentation according to the Harmonized Hippocampal Protocol.** Shown by three coronal slices of two participants. Top row: 75 years, female, e4 carrier, ADAS Cog11 score = 5; Bottom row: 70 years, female, e4 carrier, ADAS Cog11 = 10.

**Figure 2:**
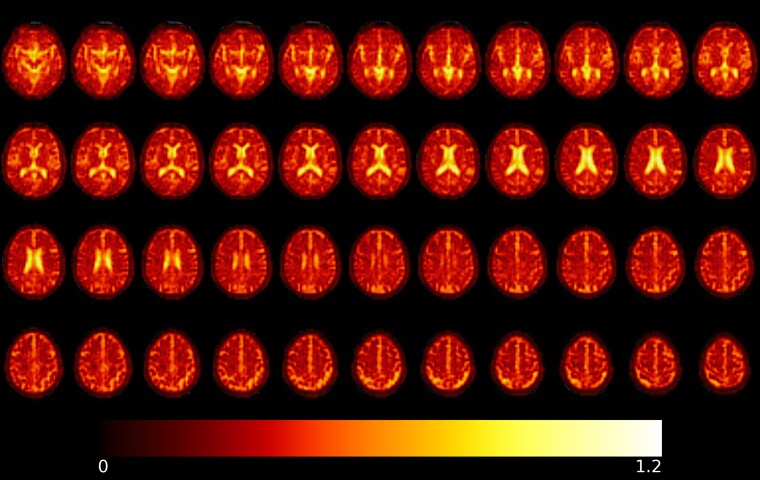
**Prenormalized sodium image.** The sodium image is B0 and B1 inhomogeneity corrected overlayed on a T1-weighted image for a 72 year-old male, apoe4 non-carrier, ADASCog11 score = 6 (range of sodium values: 0–1.2 with colour bar).

Post processing of the sodium images included B0 field inhomogeneity correction using the frequency-segmented conjugate phase reconstruction method and B1 field inhomogeneity correction using the double flip angle method on a custom-built pipeline using Matlab (Version R2015b, Natick, MA).^25^ From the proton 3D-T1-weighted images, (i) gray matter, white matter and CSF were segmented using FreeSurfer (Version 6.0)^26^; (ii) the left and right hippocampi were manually segmented by a radiology registrar (MC) under the supervision of a neuroradiologist of 15 years’ experience (EL) according to the Harmonized Hippocampal Protocol^27^ [Fig. 1]; (iii) left and right precuneus, entorhinal, cuneus (as control), and pre and post central cortices were parcellated using FreeSurfer (Version 6.0).^26^ The T1-weighted images were then co-registered to the sodium images via the T2-weighted images with Advanced Normalization Tools (version 2.4.4)^28^ [Fig. 2]. The sodium and T1 images were registered to T2 image separately and then the combined transformation was applied to the sodium image. The co-registration between T1 and sodium image were then manually checked by neuroradiologist >30 years’ experience (PD). Partial volume correction (PVC) using a hybrid Geometric Transfer Matrix–Region Based Voxel Wise (GTM-RBV) method^29,30^ resampled the sodium images to 1 mm isotropic resolution. The regions of interest (ROIs) were then extracted from the sodium images after the images and co-registrations were checked by a neuroradiologist of over 30 years’ experience (PD).

Normalized sodium signal intensity (Na-SI) of the hippocampi, entorhinal cortex and precuneus^31,32^ were obtained using the sensori-motor cortex as reference.^20,33^ The volumes of these ROIs were obtained from the T1 parcellations, normalized to intracranial volume.

### Statistical analysis

Descriptive statistics of demographics, clinical and imaging variables were obtained. Scatterplots and Spearman correlations were examined to assess relationships between variables. Best-subsets regression modelling^34^ were run for each of the three cognitive outcome measures. Linear regression was appropriate for ADAS-Cog11 with a square-root transform, and logistic regression for MMSE and CERAD. Logistic regression was appropriate for MMSE and CERAD given the ceiling effects; scores were modelled as the number of successes out of the total number of items. Each best-subsets regression included twelve candidate predictor variables, eight common predictor variables (such as age and sex) and four ROI-specific variables (for example, left and right hippocampal sodium and volumes.) These variables are fully listed in Supplementary Table 1. All possible models with up to eight predictor variables were considered. The best models were short-listed according to the Akaike Information Criterion (AIC), a smaller AIC indicating better model fit and models within an AIC value of 2 of the model with the lowest AIC were considered equivalent.^34^ The final determination was according to the principle of parsimony—amongst the models that were equivalent, the models that involved fewer explanatory variables were selected. Regression analyses of our final best-subset for each cognitive outcome were performed without and then with amyloid status added. The level of statistical significance was defined *P* < 0.05. Genstat (V19.1.0, VSN International Ltd, Hemel-Hempstead, United Kingdom) was the software used and data generated or analysed during the study are available from the corresponding author by request.

## Results

### Participant characteristics

**Figure 3:**
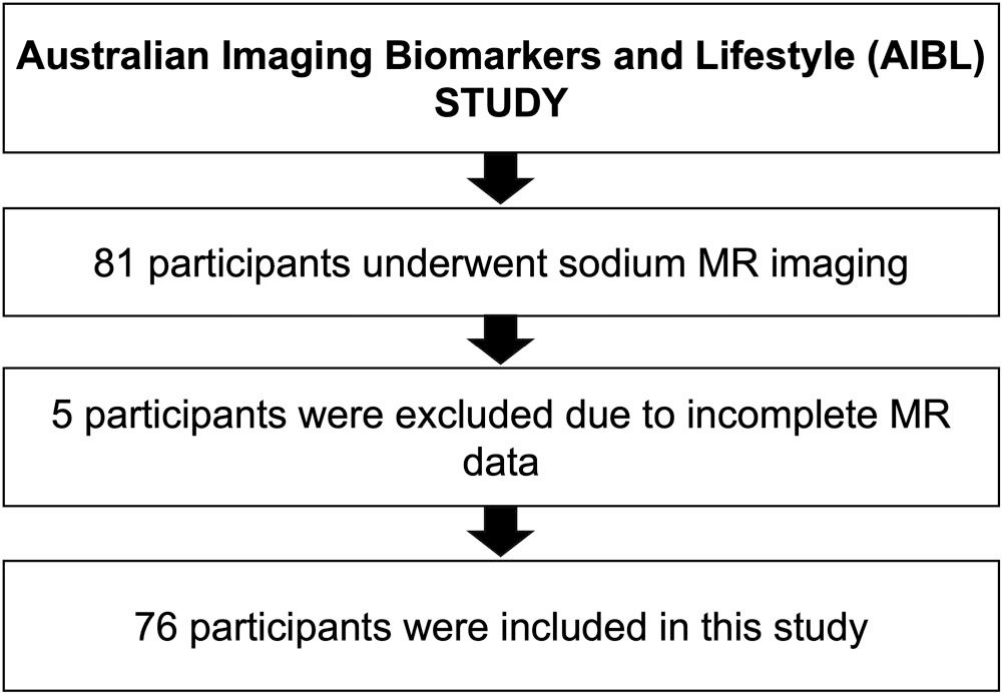
Flow diagram of participants included and excluded from this study.

Our 76 nondemented participants (after 5 were excluded for incomplete data [Fig. 3]) had a mean (SD) age of 75(5) years, 47/76(62%) woman, mean education 13 years, 19/76(25%) APOE e4 positive, 54/76(71%) cognitively unimpaired and 22/76 (29%) mildly cognitively impaired (MCI). Out of 22 MCI, 3 were amnestic multiple domain; 16 were amnestic single domain; and 3 were non-amnestic single domain MCI participants. Their mean (SD, range) scores for ADAS-Cog11, MMSE and CERAD were 7(4,24), 29(1,7) and 10(1,5) respectively. CERAD, and to a lesser degree MMSE, showed a ceiling effect. Of the 40/76(53%) of our community volunteer participants who agreed to brain amyloid PET imaging, 9(23%) were amyloid positive. For the nine participants who were amyloid positive, 6 were cognitive normal, 3 were MCI. For the 31 participants who were amyloid negative, 25 were cognitive normal, 6 were MCI.

**Figure 4:**
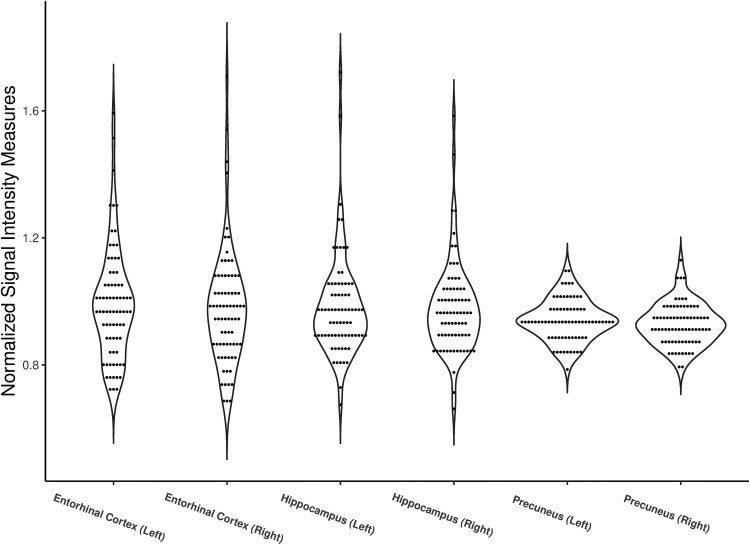
Normalized sodium values for hippocampus, entorhinal cortex, and precuneus for 76 participants.

Table 1, with Fig. 4, summarizes participant characteristics and the imaging measures of our three regions of interest (hippocampus, entorhinal and precuneus). There was no significant correlation between Na-SI and volume for the three regions of interest (Spearman correlation coefficient −0.004 to 0.136).

**Table 1:** Participant characteristics presented as median and interquartile range

Demographic and clinical variables	*N* = 76
Age	75 (7)
Sex (Woman, %)	47, 62%
Years of Education	13 (5)
APOE e4 (Positive, %)	19, 25%
Cognitive status (Cognitively unimpaired/Mild Cognitively impaired)	54/22
ADAS-Cog11 score^a^	6 (4)
MMSE score^b^	29 (2)
CERAD score^b^	10 (2)
Cerebral Amyloid Status (Positive/Negative/Unknown)	9/31/36
**Sodium signal intensity measures (normalized)**	
Left Hippocampus	0.961 (0.148)
Right Hippocampus	0.953 (0.139)
Left Entorhinal	0.981 (0.190)
Right Entorhinal	0.971 (0.200)
Left Precuneus	0.940 (0.090)
Right Precuneus	0.927 (0.090)
Volumetric measures (normalized) *1000	
Left Hippocampus	1.538 (0.250)
Right Hippocampus	1.504 (0.220)
Left Entorhinal	1.441 (0.290)
Right Entorhinal	1.271 (0.270)
Left Precuneus	5.925 (0.920)
Right Precuneus	6.108 (0.780)

^a^Higher score = worse cognition.

^b^Lower score = worse cognition.

### Normalized hippocampus sodium values (Na-SI) compared to volume

In the hippocampal analysis (Table 2), the only imaging variable selected in the best ADAS-Cog11 model was left hippocampal Na-SI (B = 1.21 (CI = 0.28, 2.14), *P* = 0.01). The other variables selected in the best model were APOE e4 (B = 0.47 (CI = 0.12, 0.82), *P* = 0.01), years of education (B = −0.05 (CI = −0.10, −0.01), *P* = 0.02) and age (B = 0.03 (CI = −0.001, 0.06), *P* = 0.054). Neither right nor left hippocampal volume was selected in the best ADAS-Cog11 model.

**Table 2:** Summary of results from the best model for hippocampus and neurocognitive measures

Variables in best model		ADAS-Cog11	MMSE	CERAD
	*Estimate*	*Regression coefficient*	*Odds ratio*	*Odds ratio*
Age^a^	*Estimate*	0.03		
	*95% CI*	−0.001, 0.06		
	*P-value*	0.054*		
ApoE e4^b^	*Estimate*	0.47		
	*95% CI*	0.12, 0.82		
	*P-value*	0.01*		
Years of Education^a^	*Estimate*	−0.05		1.11
	*95% CI*	−0.10, −0.01		1.03, 1.20
	*P-value*	0.02*		0.01*
Left Hippocampus Na-SI^a^	*Estimate*	1.21	0.19	
	*95% CI*	0.28, 2.14	0.07, 0.53	
	*P-value*	0.01*	0.001*	
*Adjusted R squared*		16.92	5.72	5.85
*Akaike Information Criteria*		81.00	78.00	78.00
Addition of cerebral amyloid status				
Cerebral amyloid status^b^	*Estimate*	0.61	0.56	0.27
	*95% CI*	0.12, 1.09	0.31, 0.99	0.13, 0.57
	*P-value*	0.02*	0.047*	<0.001*
Cerebral amyloid status^c^	*Estimate*	0.25	1.11	0.55
	*95% CI*	−0.06, 0.57	0.69, 1.79	0.31, 0.98
	*P-value*	0.11	0.67	0.04*
*Adjusted R squared*		22.29	7.27	13.81
*Akaike Information Criteria*		83.00	80.00	80.00

^a^Level: x + 1, Baseline: x.

^b^Level: Positive, Baseline: Negative.

^c^Level: Unknown, Baseline: Negative.

^*^ *P* < 0.05.

For the best MMSE model, the only variable selected was the left hippocampal Na-SI (OR = 0.19 (CI = 0.07, 0.53), *P* = 0.001). None of the hippocampal volumes and demographic variables were selected in the best MMSE model.

For the best CERAD model, education was the sole variable selected (OR = 1.11 (CI = 1.03, 1.20), *P* = 0.01). Neither hippocampal Na-SI nor volumes were selected variables in the best CERAD model.

The cuneus variables, our control ROI, were not selected by any of the three neurocognitive score best models.

### Normalized entorhinal sodium values (Na-SI) compared to volume

In the entorhinal analysis (Table 3), the only imaging variable selected in the best ADAS-Cog11 model was Na-SI of the right entorhinal ROI (B = 0.94 (CI = 0.11, 1.77), *P* = 0.03). The other variables selected in the best model were APOE e4 (B = 0.46 (CI = 0.11, 0.81), *P* = 0.01), years of education (B = −0.05 (CI = −0.10, −0.01), *P* = 0.02) and age (B = 0.03 (CI = 0.003, 0.06), *P* = 0.03). Neither right nor left entorhinal volume was selected in the best ADAS-Cog11 model.

**Table 3:** Summary of results from the best model for entorhinal cortex and neurocognitive measures

Variables in best model		ADAS-Cog1I	MMSE	CERAD
	*Estimate*	*Regression coefficient*	*Odds ratio*	*Odds ratio*
Age^a^	*Estimate*	0.03	0.96	
	*95% CI*	0.003, 0.06	0.92, 1.00	
	*P-value*	0.03*	0.04*	
ApoE e4^b^	*Estimate*	0.46		
	*95% CI*	0.11, 0.81		
	*P-value*	0.01*		
Years of Education^a^	*Estimate*	−0.05		1.11
	*95% CI*	−0.10, −0.01		1.03, 1.20
	*P-value*	0.02*		0.005*
Right Entorhinal Volume^a^	*Estimate*		2.62	
	*95% CI*		1.04, 6.57	
	*P-value*		0.04*	
Right Entorhinal Na-SI^a^	*Estimate*	0.94	0.23	
	*95% CI*	0.11, 1.77	0.09, 0.60	
	*P-value*	0.03*	0.003*	
*Adjusted R squared*		15.14	10.32	5.85
*Akaike Information Criteria*		81.00	80.00	78.00
Addition of cerebral amyloid status				
Cerebral amyloid status^b^	*Estimate*	0.60	0.48	0.27
	*95% CI*	0.11, 1.09	0.27, 0.88	0.13, 0.57
	*P-value*	0.02*	0.02*	<0.001*
Cerebral amyloid status^c^	*Estimate*	0.27	1.03	0.55
	*95% CI*	−0.05, 0.59	0.63, 1.68	0.31, 0.98
	*P-value*	0.10	0.91	0.04*
*Adjusted R squared*		20.56	13.03	13.81
*Akaike Information Criteria*		83.00	82.00	80.00

^a^Level: x + 1, Baseline: x.

^b^Level: Positive, Baseline: Negative.

^c^Level: Unknown, Baseline: Negative.

^*^ *P* < 0.05.

For the best MMSE model, three variables were selected. The right entorhinal Na-SI had the smallest *P*-value (OR = 0.23 (CI = 0.09, 0.60), *P* = 0.003), followed by age (OR = 0.96 (CI = 0.92, 1.00), *P* = 0.04), then the right entorhinal volume (OR = 2.62 (CI = 1.04, 6.57), *P* = 0.04). Independently, Na-SI explained more of the variance (*R*^2^ = 10.3) than volume (*R*^2^ = 7.5).

For the best CERAD model, like the hippocampal analysis, only education was selected (OR = 1.11 (CI = 1.03, 1.20), *P* = 0.005). Again, neither entorhinal Na-SI nor volumes were selected variables in the best CERAD model.

The cuneus variables were again not selected for all three neurocognitive score best models.

### Normalized precuneus sodium values (Na-SI) compared to volume

In the precuneus analysis (Table 4), all three imaging variables selected in the best ADAS-Cog11 model were Na-SI, which had smaller *P*-values than the demographic variables selected: left and right precuneus Na-SI (left B = 8.40 (CI = 3.51, 13.28), *P* = 0.001; right B = −9.39 (CI = −14.85, −3.93), *P* = 0.001), followed by right cuneus Na-SI (B = 2.51 (CI = 0.68, 4.33), *P* = 0.008), education (B = −0.05 (CI = −0.09, −0.003), *P* = 0.04) and APOE e4 (B = 0.35 (CI = 0.01, 0.68), *P* = 0.04).

**Table 4:** Summary of results from the best model for precuneus and neurocognitive measures

Variables in best model		ADAS-Cog11	MMSE	CERAD
	*Estimate*	*Regression coefficient*	*Odds ratio*	*Odds ratio*
Sex^a^	*Estimate*		1.68	
	*95% CI*		1.09, 2.59	
	*P-value*		0.02*	
ApoE e4^b^	*Estimate*	0.35		
	*95% CI*	0.01, 0.68		
	*P-value*	0.04*		
Years of Education^c^	*Estimate*	−0.05	1.09	1.11
	*95% CI*	−0.09, −0.003	1.02, 1.17	1.03, 1.20
	*P-value*	0.04*	0.01*	0.005*
Left Precuneus Na-SI^c^	*Estimate*	8.40		
	*95% CI*	3.51, 13.28		
	*P-value*	0.001*		
Right Precuneus Na-SI^c^	*Estimate*	−9.39	1064	
	*95% CI*	−14.85, −3.93	14.78, 76880	
	*P-value*	0.001*	0.001*	
Left Cuneus Na-SI^c^	*Estimate*		0.01	
	*95% CI*		0, 0.05	
	*P-value*		<0.001*	
Right Cuneus Na-SI^c^	*Estimate*	2.51		
	*95% CI*	0.68, 4.33		
	*P-value*	0.008*		
*Adjusted R squared*		22.13	14.81	5.85
*Akaike Information Criteria*		82.00	81.00	78.00
Addition of cerebral amyloid status				
Cerebral amyloid status^b^	*Estimate*	0.49	0.53	0.27
	*95% CI*	0.01, 0.97	0.29, 0.96	0.13, 0.57
	*P-value*	0.045*	0.035*	<0.001*
Cerebral amyloid status^d^	*Estimate*	0.18	1.07	0.55
	*95% CI*	−0.13, 0.49	0.66, 1.76	0.31, 0.98
	*P-value*	0.25	0.78	0.041*
*Adjusted R squared*		24.73	16.64	13.81
*Akaike Information Criteria*		84.00	83.00	80.00

^a^Level: Woman, Baseline: Man.

^b^Level: Positive, Baseline: Negative.

^c^Level: x + 1, Baseline: x.

^d^Level: Unknown, Baseline: Negative.

^*^ *P* < 0.05.

Similar to ADAS-Cog11, for the best MMSE model, both imaging variables selected were Na-SI and Na-SI had smaller *P*-values than the demographic variables selected: left cuneus Na-SI (OR = 0.01 (CI = 0, 0.05), *P* = <0.001), right precuneus Na-SI (OR = 1064 (CI = 15, 77000), *P* = 0.001), education (OR = 1.09 (CI = 1.02, 1.17), *P* = 0.01) and sex (OR = 1.68 (CI = 1.09, 2.59), *P* = 0.02).

Consistent with the hippocampal and entorhinal analyses, for the best CERAD model, only education was selected (OR = 1.11 (CI = 1.03, 1.20), *P* = 0.005) and no volume variable was selected in any of the three neurocognitive score models.

Further post hoc correlation and regression analyses for the precuneus ADAS-Cog11 and MMSE results are presented in Supplementary Tables 2–4.

### Cerebral amyloid PET status

Adding known positive cerebral amyloid status to the best models slightly further improved the model fit, with a consistent increase in AIC of 2.0 for across all neurocognitive test models for all three ROIs (*P* < 0.001–0.045) (Tables 2–4).

## Discussion

With the urgent need for effective disease modifying therapy (DMT) to combat the increasing burden of dementia affecting an ageing population world-wide, we evaluated, in a nondemented older adult cohort, whether regional normalized sodium-MRI (Na-SI) may be a better predictor of neurocognitive status than volumetry to potentially complement existing neurocognitive test trial endpoints. We showed (i) Na-SI was consistently the only imaging variable selected across all three brain regions investigated for predicting ADAS-Cog11 (0.001 < *P* < 0.03); (ii) left hippocampal Na-SI was the only variable (out of 12 candidate variables, including clinical variables) selected for predicting MMSE scores (*P* = 0.001); (iii) neither Na-SI nor volume was selected for CERAD; (iv) slight further improvement of the regression model fit when cerebral amyloid PET positive status was known (AIC improved by 2.0 for all three regions and across all three neurocognitive scores), even in our small subgroup of 9/40 amyloid positive participants. Overall, these findings support our hypothesis of Na-SI being a more predictive MRI metric for neuronal/cognitive function than atrophy in even the normal to mildly cognitively impaired older adult setting.

Our method was built on the strengths of a variety of existing work, both within and outside of the sodium-MRI literature, to suit a clinical scanning environment and to be practicable for clinical trial settings. We used twisted projection imaging at 90-degree flip angle, ultrashort TE, with both B0 and B1 inhomogeneity corrections.^35^ We adapted from PET (of similar resolution to 3T sodium-MRI) two techniques for partial volume correction^36^ which, as evidenced by our previous phantom and in vivo studies, significantly improved accuracy.^29,30^ In the studied phantom, the discrepancy between the measured and expected sodium concentration was reduced from 11% to 5%.^29,30^ For the in vivo study, the PVC algorithm reduced the mean discrepancy in two separate CSF compartments from 36% to 7%.^26^ Thus, our PVC method effectively reduced partial volume effect-induced error to a range of about 5–7% and this estimate aligns with findings from an independent report by Niesporek *et al*.^37^ We improved from Mellon *et al.*’s lateral ventricle ROI for normalization after yielding a better reference ROI in the sensorimotor cortex from our previous work^13,33^ to obtain a Na-SI metric that does not require phantom-based quantitation. While there is literature showing sensorimotor cortex to be a superior reference region for PET normalization in Alzheimer’s disease,^20^ sensorimotor cortex reference region for sodium signal normalization is novel. In our previously published work on reference region selection for sodium signal normalization,^33^ we found that sensorimotor cortex and midbrain were both suitable reference regions. We proceeded to select sensorimotor cortex as our reference region because a sizeable number of our older adult cohort had small vessel ischaemic change and prominent perivascular spaces in the midbrain. Despite low signal to noise in the lower brainstem structures in our dataset precluding using whole brainstem for normalization, we demonstrated no significant difference (*P*-values 0.3–0.5) in Na-SI using sensorimotor cortex compared to midbrain as reference regions in our cohort of 54 cognitively normal participants (Supplementary Table 5), validating the use of sensorimotor cortex reference region for sodium signal normalization as an alternative to the brainstem as published by Haegar *et al*.^16^ Our hippocampal segmentations were according to the Harmonized Protocol, the global reference standard for MRI manual hippocampal segmentation^27^ since Mellon *et al*. We included two software-segmented ROIs, entorhinal and precuneus cortices, known areas of interest in early Ad that avoid confounding contribution of white matter disease and perivascular spaces (both prevalent in older adults) to Na-SI, compared to Mohamed *et al.*’s lobar cortical^14^ and Haeger *et al.*’s ROIs that included white matter.^15,16^ All four sodium-MRI Ad studies to date combined left and right ROIs,^13-16^ but due to the hippocampal lateralization differences found by Thulborn *et al.*,^17^ we remained left-right separated.

Our cohort extends the sodium-MRI literature in normal ageing and pre-dementia and our study has extended the evaluation of sodium-MRI for this cohort beyond group comparisons^38^ to specifically evaluate relationships between Na-SI values and neurocognitive test score metrics in continuum in the nondemented older adult space. Consistent with established understanding of the biological basis of altered brain tissue sodium concentration, neural dysfunction due to deranged cellular metabolism-energetics leading to dysfunction of the sodium-potassium ATPase pump no longer being able to maintain the transmembrane sodium-potassium gradient leading to an influx of extracellular sodium into the intracellular compartment to elevate the overall tissue sodium concentration according to the Eq. 1 in Thulborn,^17^ we have demonstrated elevated Na-SI with worse neurocognitive test scores. A cohort of 76 is large for sodium-MRI studies. The composition of our cohort consisted of nondemented older adult participants (>60 years, range 62–90 years, 54/76 cognitively unimpaired, 22/76 MCI), compared to Thulborn *et al.*’s normal ageing study where participants ranged from 21–80 years^17^ and the Ad studies that recruited up to 22 aged-controls and two MCI participants.^13-16^ Our cohort is of interest in dementia trials because interventions are likely to be most beneficial in the predementia stages, but also the cohort that challenges volumetry, a macroscopic metric. The distribution of our neurocognitive test scores reflected our cohort and paralleled our results: (i) most consistent in the most broadly & normally spread ADASCog-11 scores, (ii) mainly consistent for MMSE where there was some ceiling effect and (iii) no imaging predictor with CERAD’s strong ceiling effect and combination of narrow spread and range. The strong ceiling effect with narrow spread and range of the CERAD scores in our cohort is the most likely explanation for neither volumes nor Na-SI predictive for CERAD. This is particularly as our CERAD scores are of the CERAD-praxis measure that screen for visuospatial and constructional abilities^39^ and 19/22 of our MCI participants are amnestic, 16/19 are amnestic single domain. Therefore, although the precuneus is known to be involved in visuospatial tasks,^40^ our participants are relatively mildly impaired in this domain (mean CERAD-praxis score 10 compared to 5.4–7.3 in a dementia cohort where FDG-PET showed significant correlation between CERAD-praxis score and biparietal atrophy^39^). This is further supported by another study that evaluated the relationship between MRI cortical thickness and prodromal Ad (98 controls, 100 MCI with 22/100 converting to Ad within 1 year having mean CERAD-praxis score 9.7–10.4) where no significant cortical thinning was found in the parietal lobes, including the precuneus.^41^

A positive amyloid PET status improving fit across all best models, despite the low number of known amyloid PET positive participants of 9/40 in our observational community volunteer cohort, is promising pilot results for potential Ad applications. This is unsurprising, as our ROIs of hippocampi, entorhinal cortex and precuneus were specifically selected as known regions early pathological involvement in AD.^31,32^ Furthermore, our results of the hippocampi closely followed by the entorhinal cortex as the most robust ROIs likely reflect the high number of (19/22) MCI participants of the amnestic type irrespective of the sizeable number of participants (36/76) with unknown amyloid PET status.

Our findings in this cohort demonstrate the capability of sodium-MRI and reveal potential applications beyond primarily identifying group differences between established Ad (with mean MMSE ranging from 20 to 25) and controls.^13-15^ It is remarkable that, even at the widely available 3T field-strength, Na-SI was predictive of subtle differences in neurocognition within our nondemented older adult participants with high mean MMSE of 29 and low SD of 1 (MMSE is out of 30). This extends from Haeger *et al.*’s findings of sodium concentrations being predictive of the Montreal Cognitive Assessment score in their two studies, one spanning controls to Ad patients with mean MMSE of 20 at 7T^15^ and another evaluating the Ad subgroup at 3T.^16^ While our manual gold-standard hippocampal segmentation may have contributed to the robustness of the hippocampal ROI compared to entorhinal cortex, incomplete partial volume correction may also be contributory. However, our positive findings for both hippocampus and entorhinal cortex are unsurprising. Mesio-temporal areas are affected by tau accumulation early in the neuropathology of Ad that corresponds to the typical amnestic phenotype^31,42^ and pathological tau (that causes neuronal dysfunction with resultant cognitive decline) is known to accumulate in neuronal bodies and dendrites which are structures in the cortex^43^; memory is an important component of Ad neurocognitive assessments; and mesio-temporal brain tissue sodium perturbations secondary to impaired sodium-potassium ATPase pump function from neuronal metabolic derangement increasing the intracellular concentration of sodium to result in an increase in overall brain tissue sodium concentration^17^ are consistent with the previous sodium-MRI Ad studies.^13-15^

Our left-right separation may have also increased sensitivity to detect the subtle differences in neurocognition in our cohort by reducing the smoothing effect of averaging. Our results furthermore suggest segmenting just the left hippocampus may be sufficient. The resource saving implications of this in the context of clinical trials is significant, a decrease of resource-intensive manual segmentation time by 50%.

Our results suggest Na-SI may have broader applications beyond Ad. While ADASCog-11 was specifically designed for AD^44^ and the gold standard for Ad DMT evaluation,^45^ MMSE is widely used clinically and in research outside of Ad. In our cohort where at least 31/76 of our participants are PET amyloid negative, left hippocampal Na-SI was impressively the sole variable chosen for MMSE prediction.

In contrast, our precuneus results are less convincingly predictive. While a very strong correlation between right and left precuneus is unsurprising, the strong correlation between right precuneus and left cuneus Na-SI is unclear, but explainable by Nelson *et al.*’s findings of the precuneus not being more affected by Ad pathology than other neo-cortex despite abundant morphological and functional imaging findings of the precuneus being affected early.^46^ Nevertheless, Na-SI remains the only imaging metric selected, despite evidence of interactions between strongly correlated predictor variables, consistent with Na-SI being the better predictor variable over volume in even the precuneus.

Our study’s main limitation is 36/76 of our nondemented community volunteers was of unknown cerebral amyloid status, and of the 40 who consented to amyloid PET scanning, only nine were amyloid positive. Future research on an amyloid-positive enriched cohort would be worthwhile, whether outside or within of the context of an exploratory endpoint in DMT trials where MRI would be used for baseline and DMT side-effect monitoring anyway. Another limitation is the small number of MCI participants with the high ceiling effect of the neurocognitive test scores from predominantly cognitive normal participants preclude meaningful separate within-group modelling of sodium-MRI values versus the neurocognitive test scores. Similarly, the sample size limited the number of explanatory variables that could be included in our models, hence our best subsets regression approach for each of our selected ROIs in this study. Further evaluation of multiple ROIs under a unified model with a larger dataset would be interesting to explore the contributions of different regions to cognitive performance.

Concluding, regional Na-SI were more predictive of ADAS-Cog11 and MMSE scores in our nondemented older adult cohort than volume, hippocampal Na-SI more robust than entorhinal cortex Na-SI. Sodium MRI may have applications as a non-invasive imaging neurocognitive marker.

## Supplementary Material

fcae307_Supplementary_Data

## Data Availability

Data generated or analysed during the study are available from the corresponding author by request.
