# Supplementary material for: 3T sodium-MRI as predictor of neurocognition in nondemented older adults: a cross sectional study
Source: Brain Commun. 2024 Sep 11;6(5):fcae307. doi: 10.1093/braincomms/fcae307 (PMC11420980; doi:10.1093/braincomms/fcae307)
Supplement: fcae307_Supplementary_Data [file fcae307_supplementary_data.docx]

**Supplementary Material**

**Supplementary Table 1: Candidate predictor variables in best subsets regression models of ADAS-Cog11, MMSE and CERAD**

|  | **Hippocampus** | **Entorhinal** | **Precuneus** |
| --- | --- | --- | --- |
|  | ***Common explanatory variables*** | | |
|  | (1) Age, (2) APOE e4, (3) Years of Education, (4) Sex, (5) Left Cuneus Volume,  (6) Right Cuneus Volume, (7) Left Cuneus Na-SI, (8) Right Cuneus Na-SI | | |
|  | ***Specific explanatory variables*** | | |
| 9 | Left Hippocampus Na-SI | Left Entorhinal Na-SI | Left Precuneus Na-SI |
| 10 | Right Hippocampus Na-SI | Right Entorhinal Na-SI | Right Precuneus Na-SI |
| 11 | Left Hippocampus Volume | Left Entorhinal Volume | Left Precuneus Volume |
| 12 | Right Hippocampus Volume | Right Entorhinal Volume | Right Precuneus volume |

Volumes are normalized to intracranial volume

**Supplementary Table 2: Spearman correlation of sodium Na-SI measure for precuneus and cuneus ROIs.**

|  | ***Left Precuneus*** | ***Right Precuneus*** | | ***Left Cuneus*** | ***Right Cuneus*** | |
| --- | --- | --- | --- | --- | --- | --- |
| ***Left Precuneus*** | 1.00 | 0.88^*^ | 0.62^*^ | | | 0.58^*^ |
| ***Right Precuneus*** |  | 1.00 | 0.71^*^ | | | 0.69^*^ |
| ***Left Cuneus*** |  |  | 1.00 | | | 0.83^*^ |
| ***Right Cuneus*** |  |  |  | | | 1.00 |

*P<0.001

**Supplementary Table 3: Post hoc analysis to further explore the best model for precuneus ROI and ADAS-Cog11 neurocognitive measure.**

| **Variables in best model** |  | ***MODEL 1***  **ADAS-Cog11** | ***MODEL 2***  **ADAS-Cog11** |
| --- | --- | --- | --- |
|  | ***Estimate*** | ***Regression coefficient*** | ***Regression coefficient*** |
| **ApoE e4^a^** | *Estimate* | 0.39 | 0.35 |
|  | *95% CI* | 0.03, 0.75 | -0.01, 0.71 |
|  | *P-value* | 0.03^*^ | 0.06 |
| **Years of Education^b^** | *Estimate* | -0.05 | -0.05 |
|  | *95% CI* | -0.09, -0.002 | -0.10, -0.01 |
|  | *P-value* | 0.042^*^ | 0.02^*^ |
| **Left Precuneus Na-SI^b^** | *Estimate* | 1.39 |  |
|  | *95% CI* | -1.51, 4.29 | - |
|  | *P-value* | 0.34 |  |
| **Right Precuneus Na-SI^b^** | *Estimate* |  | -1.57 |
|  | *95% CI* | - | -4.81, 1.66 |
|  | *P-value* |  | 0.34 |
| **Right Cuneus Na-SI^b^** | *Estimate* | 0.92 | 2.01 |
|  | *95% CI* | -0.77, 2.61 | 0.07, 3.94 |
|  | *P-value* | 0.28 | 0.042^*^ |
| ***Adjusted R squared*** |  | *10.33* | *10.35* |
| ***Akaike Information Criteria*** |  | *91.77* | *91.74* |
| **Addition of cerebral amyloid status** | |  |  |
| **Cerebral amyloid status^a^** | *Estimate* | 0.58 | 0.58 |
|  | *95% CI* | 0.08, 1.09 | 0.07, 1.09 |
|  | *P-value* | 0.02^*^ | 0.03^*^ |
| **Cerebral amyloid status^c^** | *Estimate* | 0.23 | 0.22 |
|  | *95% CI* | -0.10, 0.55 | -0.11, 0.55 |
|  | *P-value* | 0.17 | 0.19 |
| **Adjusted R squared** |  | *14.62* | *14.77* |
| **Akaike Information Criteria** |  | *92.28* | *92.14* |

^a^ Level: Positive, Baseline: Negative

^b^ Level: x+1, Baseline: x

^c^ Level: Unknown, Baseline: Negative

^*^ P<0.05

**Supplementary Table 4: Post hoc analysis to further explore the best model for precuneus ROI and MMSE neurocognitive measure.**

| **Variables in best model** |  | ***Model 1***  **MMSE** | ***Model 2***  **MMSE** | |  |
| --- | --- | --- | --- | --- | --- |
|  | ***Estimate*** | ***Odds ratio*** | ***Odds ratio*** | |  |
| **Sex^a^** | *Estimate* | 1.60 | 1.53 | |  |
|  | *95% CI* | 1.04, 2.46 | 1.00, 2.34 | |  |
|  | *P-value* | 0.03^*^ | 0.05^*^ | |  |
| **Years of Education^b^** | *Estimate* | 1.05 | 1.06 | |  |
|  | *95% CI* | 0.99, 1.12 | 0.99,1.13 | |  |
|  | *P-value* | 0.12 | 0.06 | |  |
| **Right Precuneus Na-SI^b^** | *Estimate* | 2.58 |  | |  |
|  | *95% CI* | 0.10, 67.41 | - | |  |
|  | *P-value* | 0.57 |  | |  |
| **Left Cuneus Na-SI^b^** | *Estimate* |  | 0.07 | |  |
|  | *95% CI* | - | 0.01, 0.42 | |  |
|  | *P-value* |  | 0.004^*^ | |  |
| ***Adjusted R squared*** |  | *1.10* | *7.42* | |  |
| ***Akaike Information Criteria*** |  | *91.59* | *86.24* | |  |
| **Addition of cerebral amyloid status** | |  | |  | |
| **Cerebral amyloid status^c^** | *Estimate* | 0.49 | 0.54 | |  |
|  | *95% CI* | 0.27, 0.88 | -1.21, -0.03 | |  |
|  | *P-value* | 0.02^*^ | 0.04^*^ | |  |
| **Cerebral amyloid status^d^** | *Estimate* | 1.14 | 1.05 | |  |
|  | *95% CI* | 0.69, 1.87 | -0.44, 0.54 | |  |
|  | *P-value* | 0.61 | 0.84 | |  |
| ***Adjusted R squared*** |  | *4.44* | *8.81* | |  |
| ***Akaike Information Criteria*** |  | *92.25* | *88.57* | |  |

^a^ Level: Woman; Baseline: Man

^b^ Level: x+1; Baseline: x

^c^ Level: Positive; Baseline: Negative

^d^ Level: Unknown; Baseline: Negative

^*^ P<0.05

**Supplementary Table 5: Comparison of normalised Na SI measures by sensori-motor cortex and midbrain in cognitively normal (N=54)**

|  | **Normalised with sensori-motor cortex** | **Normalised with midbrain** | **Paired T-Test (p-values)**  **N=54** |
| --- | --- | --- | --- |
| **Left Hippocampus** | 0.9647 (0.1476) | 0.9845 (0.1912) | 0.292^#^ |
| **Right Hippocampus** | 0.9647 (0.1499) | 0.9721 (0.1929) | 0.293 |
| **Left Precuneus** | 0.9408 (0.0935) | 0.9282 (0.2303) | 0.476 |
| **Right Precuneus** | 0.9300 (0.1059) | 0.9137 (0.2479) | 0.453 |
| **Left Entorhinal Cortex** | 0.9771 (0.2067) | 0.9841 (0.1977) | 0.245 |
| **Right Entorhinal Cortex** | 0.9798 (0.2195) | 0.9491 (0.1951) | 0.229 |

# Related-Samples Wilcoxon Signed Rank Test
